# Taohong Siwu Decoction Exerts a Beneficial Effect on Cardiac Function by Possibly Improving the Microenvironment and Decreasing Mitochondrial Fission after Myocardial Infarction

**DOI:** 10.1155/2019/5198278

**Published:** 2019-12-10

**Authors:** Zhi-rong Luo, Han Li, Zhong-xin Xiao, Shui-jin Shao, Tian-tian Zhao, Yue Zhao, Fang-fang Mou, Bo Yu, Hai-dong Guo

**Affiliations:** ^1^Department of Anatomy, School of Basic Medicine, Shanghai University of Traditional Chinese Medicine, Shanghai 201203, China; ^2^Department of Cadre's Cardiovascular, The First Affiliated Hospital of Anhui University of Traditional Chinese Medicine, Anhui 230031, China; ^3^Department of Neurological Rehabilitation, The Second Rehabilitation Hospital of Shanghai, Shanghai 200441, China

## Abstract

Cardiovascular disease has been established as a major cause of morbidity and mortality worldwide, resulting in a huge burden to patients, families, and society. Traditional Chinese Medicine (TCM) presents several advantages for the prevention and treatment of cardiovascular diseases including multitargets, multi-ingredients, fewer side effects, and low cost. In this study, a rat model of myocardial infarction (MI) was established by ligating the anterior descending branch of the left coronary artery, and the effect of the Taohong Siwu decoction (THSWD) on cardiac function was evaluated in MI rats. Following the intragastric administration of THSWD, the cardiac function was examined using echocardiography. The infarct size and collagen deposition in the infarct area were measured using Masson's trichrome staining, and the number of CD31- and *α*-SMA-positive blood vessels in the peri-infarct and infarct area was evaluated by immunofluorescent staining. The mRNA expression of bFGF, IGF-1, and HGF was detected using RT-PCR assay. Cell apoptosis in the infarcted area was assessed by TUNEL staining, and the p-Akt level was detected using the western blot assay. The mitochondrial ROS production was measured using MitoSOX staining, and mitochondrial dynamics and mitophagy were evaluated with western blotting 7 days after THSWD treatment. THSWD increased the ejection fraction (EF) and fractional shortening (FS) values in the rat hearts; however, no statistical difference was found between the THSWD and MI groups 4 weeks after treatment. Furthermore, THSWD significantly decreased the value of the left ventricular end-systolic volume (LVESV). Compared with the model group, THSWD significantly increased the expression of IGF-1 and bFGF, reduced collagen deposition, promoted angiogenesis, reduced cell apoptosis, and activated the PI3K/Akt signaling pathway. Notably, THSWD significantly decreased mitochondrial ROS production and Fis1 expression. No statistical differences were observed in the expression of mitochondrial LC3B and Mfn1 between the THSWD and control groups. In summary, THSWD may possess a beneficial effect on cardiac function by improving the local ischemic microenvironment and by decreasing mitochondrial fission after MI. Hence, this may present a promising auxiliary strategy in the treatment of ischemic cardiomyopathy such as MI.

## 1. Introduction

Cardiovascular disease has been established as a major cause of morbidity and mortality worldwide, introducing a huge burden to patients, families, and society. Myocardial infarction (MI) is a serious cardiovascular event that results mostly due to the occlusion of a coronary artery, preventing blood flow to the myocardium. Severe interruption of the myocardial blood supply can cause regional myocardial ischemia and hypoxia, leading to apoptosis and necrosis, followed by collagen deposition and scar tissue formation in the infarcted area, resulting in decreased myocardial contractility and heart failure [1, 2]. To date, heart failure is the leading cause of hospitalization and death and currently the greatest killer worldwide, with a survival rate of only approximately 50% at 5 years.

Adequate reperfusion of the occluded coronary artery, including percutaneous coronary interventions or thrombolysis, has been shown to limit the infarct size. Currently, early and complete reperfusion is the only accepted clinical approach to reduce cardiomyocyte injury after MI. However, reperfusion of the ischemic myocardium may cause further myocardial damage named myocardial ischemia-reperfusion injury. This is a complex process involving inflammation, oxidative stress, intracellular Ca^2+^ overload, and the opening of the mitochondrial permeability transition pores and inflammation [3]. Pharmacological drugs such as angiotensin receptor blockers, angiotensin-converting enzyme inhibitors, *β*-adrenergic receptor blockers, and statins provide symptomatic relief after MI; however, they have been found to be ineffective in repairing necrotic cardiomyocytes because cardiomyocytes undergo terminal differentiation soon after birth [4]. Ultimately, the only treatment for post-MI heart failure is heart transplantation. Nevertheless, this application is limited due to a lack of donors and the possibility of immune rejection. Therefore, it is imperative to identify potential pharmacotherapies in the treatment of MI to maintain cardiac function and structure.

There is a growing interest in the use of Traditional Chinese Medicine (TCM) to prevent and treat cardiovascular diseases. Notably, TCM has several advantages, such as multitargets, multi-ingredients, fewer side effects, and low cost. In recent decades, a large number of studies have been performed to evaluate the effectiveness and safety of TCM in the treatment of MI. Traditional Chinese herbal compounds could inhibit apoptosis and inflammation of cardiomyocytes, promote myocardial angiogenesis, mitigate cardiac dysfunction, and attenuate cardiac remodeling [5–8]. Moreover, Chinese herbal intravenous preparations may effectively lower the mortality rate of AMI in the clinics [9]. Danhong injection (DHI) is a compound injection of Chinese patent medicine composed of the roots of Danshen (*Radix Salvia Miltiorrhizae*) and the flower of Honghua (*Flos Carthami*). DHI is widely used in the treatment of acute MI in China [10, 11]. However, since the safety of DHI is yet to be fully verified, its benefits should be cautiously considered [12].

Taohong Siwu decoction (THSWD) is a famous TCM prescription and is widely used in promoting blood circulation and eliminating blood stasis and is composed of *Semen Persicae*, *Flos Carthami*, *Angelica Sinensis*, *Radix Paeoniae Alba*, *Rhizoma Chuanxiong*, and *Radix Rehmanniae Praeparata* in fixed proportions. THSWD was first recorded in a well-known medical book *Yizong Jinjian* (*Golden Mirror of Medicine*) compiled by Wu Qian in the Qing Dynasty. It has been indicated that THSWD comprised abundant drug-like and lead-like compounds that may act as potential inhibitors at numerous important target proteins associated with osteoarthritis. Furthermore, the “target-disease network” revealed that THSWD may be potentially effective in the treatment of 69 diseases, including heart failure, ischemia-reperfusion injuries, ischemic heart disease, MI, and stroke [13]. In fact, the combination of THSWD and low-dose tissue-type plasminogen activator decreased the infarct volume in cerebral ischemic regions, increased cerebral blood flow, and reduced apoptosis after embolic stroke [14]. Another study suggested that THSWD could improve the microcirculation in the femoral head in rabbits by promoting the expression of vascular endothelial growth factor (VEGF) [15]. In clinical practice, THSWD combined with conventional treatment demonstrated a potential benefit in relieving angina pectoris without significant adverse events [16]. However, to the best of our knowledge, the efficacy and safety of THSWD in MI therapy remains unelucidated.

In this study, we investigated the effect of THSWD on the changes in cardiac function, infarct size, blood vessel density, cell apoptosis, and collagen deposition in the MI rats. Furthermore, the expression of prosurvival and proangiogenesis cytokines, activation of Akt, and the effects on mitochondrial dynamics and mitophagy were examined to explore the mechanisms of THSWD treatment.

## 2. Materials and Methods

### 2.1. Myocardial Infarction Model

Thirty Sprague Dawley (SD) rats, weighing 180–200 g were obtained from the Experimental Animal Center of the Shanghai University of Traditional Chinese Medicine. The SD rats were anesthetized with 1% pentobarbital sodium and fixed in a supine position on the surgical plate. After successful endotracheal intubation, the endotracheal tube was connected to a rodent ventilator (Harvard Apparatus, USA). The skin was cut approximately 1.0 cm above the xiphoid-sternal junction, and the skin and muscles were separated. After distracting the ribs with a distractor, the heart was exposed, and the left anterior descending coronary artery was ligated 2∼3 mm below the left atrial appendage. After the ligation area and the apex of the heart turned white, the incision was sutured. The groups were assigned randomly using SPSS Statistics version 23.0. This study was approved by the Animal Ethics Committee of the Shanghai University of TCM and the Animal Research Committee of Shanghai. All experimental procedures and protocols were performed in accordance with the “Guide for the Care and Use of Laboratory Animals” of the National Institutes of Health (USA).

### 2.2. Intragastric Administration of THSWD

Two days after the establishment of MI, four rats died or failed to present the disease modeling. The remaining rats were divided into two groups: MI control group (*n* = 13) and THSWD group (*n* = 13). Eight rats in the THSWD group were intragastrically administered THSWD for 4 weeks (1 ml/day). THSWD in each rat contained *Semen Persicae* (0.16 g), *Flos Carthami* (0.16 g), *Angelica Sinensis* (0.22 g), *Radix Paeoniae Alba* (0.18 g), *Rhizoma Chuanxiong* (0.14 g), and *Radix Rehmanniae Praeparata* (0.27 g) provided by the Shuguang Hospital affiliated to the Shanghai University of Traditional Chinese Medicine (Shanghai, China). The extraction procedure for THSWD was as follows. First, the crude herbal drugs were mixed with distilled water and soaked for 30 min. Next, the mixture was extracted with boiling water for 30 min. The residue was extracted using boiling water for 20 min and subsequently filtered through four layers of gauze. Next, the two filtrates were mixed and evaporated using rotary evaporation under vacuum at 60°C and concentrated to an equivalent 1.13 g/mL of the crude herbal drugs. The dosage of THSWD was determined based on the body area according to the conversion from the human dose to rat dose. The human dosage of each component of THSWD was an effective dose commonly used in clinical practice for cardiovascular disease. Eight rats in the model group were administered an equivalent amount of intragastric physiological saline for four weeks. Five rats in each group were killed at seven days after THSWD treatment to evaluate cell apoptosis and mitochondrial function, dynamics, and mitophagy.

### 2.3. Echocardiography

The rats were anesthetized with 2% isoflurane and examined using a commercial echocardiography system (Vevo Visualsonics 2100, VisualSonics, Toronto, ON, Canada) on day 2 and 4 weeks after MI. The heart was observed along the parasternal long-axis, and each measurement was obtained in the M mode with data from an average of three consecutive cardiac cycles. The ejection fraction (EF) and fractional shortening (FS) of LV, expressed as percentages, were automatically calculated by the echocardiography software according to the Teicholz formula. Left-ventricular end-systolic (LVESV) and end-diastolic volume (LVEDV) were also measured and recorded. The operator who performed echocardiography was blinded to the animal treatments.

### 2.4. Masson's Trichrome Staining

After the cardiac function assessment, the hearts were rapidly excised and cut into three transverse slices from the base to the apex of the heart. Each slice was fixed in 4% paraformaldehyde, embedded in the optimal cutting temperature (OCT) compound (Sakura, USA), and cut into 10 *μ*m sections using a freezing microtome. Frozen sections from each group were washed using the phosphate buffer solution (PBS, 0.01 M, pH 7.2∼7.4) and stained with Masson's trichrome in accordance with the manufacturers' recommended protocol. The infarct size was defined as the sum of the epicardial and endocardial infarct circumference divided by the sum of the total LV epicardial and endocardial circumferences; the collagen content was determined by measuring the ratio of the blue area to the total area. Each parameter was quantitatively evaluated using the Image-Pro Plus software (Bethesda, USA).

### 2.5. Immunofluorescence Staining

Frozen sections from each group were washed twice with 0.01 M PBS. Next, 0.5% TritonX-100 was added to rupture the cell membrane and increase cell membrane permeability at room temperature for 15 min. After washing three times in 0.01 M PBS, the sections were blocked with normal goat serum at 37°C for 30 min to block the nonspecific binding sites. Next, the sections were incubated with the primary antibody CD31 (Abcam, 1 : 100) or *α*-SMA (Abcam, 1 : 200) at 4°C overnight and washed three times with 0.01 M PBS. The sections were then incubated with a secondary antibody coupled with Alexa Fluor 488 (1 : 200; Invitrogen, USA) at 37°C for 1 h. After washing three times with 0.01 M PBS, the sections were mounted in the antifade mounting medium, observed, and photographed under a fluorescence microscope (IX53, Olympus).

### 2.6. RT-PCR Assay

After dissection, the total RNA of the infarcted heart tissue was extracted using Trizol (Roche). The RNA concentration and purity were measured using a microplate reader (Synergy H1, BioTek, USA). RNA reverse transcription was performed using Reverse Transcriptase (Takala) in accordance with the manufacturer's instructions. PCR amplification was conducted using a PCR amplification kit (Takara) with gene-specific primers of basic fibroblast growth factor (bFGF), insulin-like growth factor-1 (IGF-1), and hepatocyte growth factor (HGF). The sequences of the PCR primer pairs (5′ to 3′) used for each gene are as follows: bFGF, AGCGGCTCTACTGCAAGAAC (forward) and TCGTTTCAGTGCCACATACC (reverse); IGF-1, GAGCGCACCTCCAATAAAGA (forward) and TCAGCGGAGCACAGTACATC (reverse); HGF, TATTGCCCTATTTCCCGTTG (forward) and GTTTCTCCTCGCCTCTCTCA (reverse). Amplification of *β*-actin from the same amount of cDNA was used as an endogenous control. Agarose gel electrophoresis at 135 V for 45 min was carried out to analyze the products, and the relative amount of each target gene was normalized to the expression of *β*-actin.

### 2.7. Terminal dUTP Nick-End Labeling Assay

To investigate the role of THSWD in cytoprotection after MI, the number of apoptotic cells was determined on day 7 after THSWD treatment using the terminal deoxynucleotidyl transferase-mediated dUTP nick-end labeling (TUNEL) staining kit (Roche, Mannheim, Germany) in accordance with the manufacturer's instructions. The nuclei were counterstained with DAPI. The total number of TUNEL-positive nuclei was counted in at least five high-power fields (HPF).

### 2.8. Western Blot Assay

The infarcted heart tissue was homogenized in ice-cold radio-immunoprecipitation assay (RIPA) lysis buffer, containing a protease inhibitor cocktail and phosphatase inhibitor (Thermo). The supernatant was collected after centrifugation at 12,000 rpm for 5 min. The mitochondria were isolated to test the effect of THSWD on mitochondrial dynamics and mitophagy. The protein concentration was measured using the BCA protein assay kit (Pierce, Rockford, IL). Next, the samples were mixed with loading buffer and denatured at 98°C for 5 min. Subsequently, an equal amount of the total protein was separated using sodium dodecyl sulfate-polyacrylamide gel electrophoresis (SDS-PAGE) and then transferred to a polyvinylidene fluoride (PVDF) membrane (Millipore, USA). The membranes were blocked with 5% skim milk PBS-T (0.1% Tween 20) on a shaker at room temperature for 1 h and incubated with the primary antibodies specific for p-Akt (CST, 1 : 1000), Akt (CST, 1 : 1000), LC3B (CST, 1 : 1000), Mfn1 (Abcam, 1 : 500), and Fis1 (Abcam, 1 : 1000) on a shaker at 4°C overnight. The primary antibodies were then identified using the horseradish peroxidase (HRP)-conjugated secondary antibody diluted 1 : 5000 (CST), followed by washing three times with 0.01 M PBS-T. Finally, the membranes were developed using an enhanced chemiluminescence (ECL) advance detection kit (Thermo). The band density was analyzed using ImageJ software (NIH, Bethesda, MD, USA).

### 2.9. Mitochondrial ROS Generation

On day 7, after THSWD treatment, MitoSOX Red (molecular probes) was used to evaluate the generation of mitochondrial superoxide as previously described [17].

### 2.10. Statistical Analysis

Values are expressed as means ± standard deviation. Statistical analysis was performed with IBM SPSS Statistics version 23.0. The statistical differences between groups were analyzed using the unpaired Student's *t*-test. Differences with a value of *P* < 0.05 were considered statistically significant.

## 3. Results

### 3.1. Effect of Taohong Siwu Decoction on Cardiac Function in MI Rats

**Figure 1:**
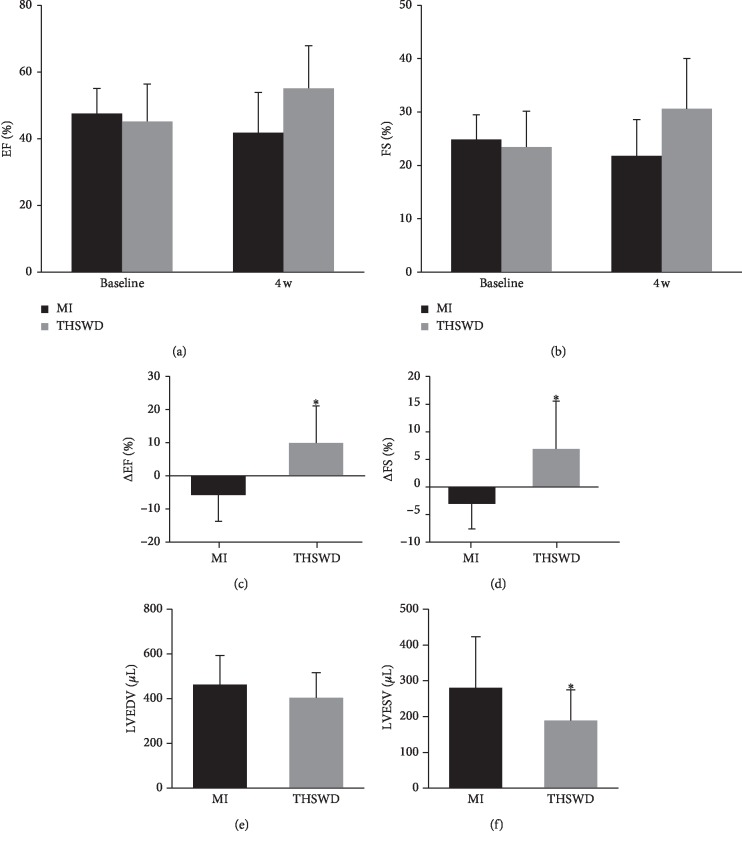
Effect of THSWD on changes in cardiac function after MI. The parameters of EF (a), FS (b), ΔEF (c), ΔFS (d), LVEDV (e), and LVESV (f) were compared between the two groups. ^*∗*^*P* < 0.05 versus the MI group.

Echocardiography was used to detect the changes in the cardiac function of MI rats four weeks after the intragastric administration of THSWD. THSWD treatment increased EF and FS values, with no statistical differences observed between the THSWD group and MI group (Figures 1(a), and 1(b)). After analyzing the values at baseline (before treatment with THSWD) and 4 weeks after treatment, EF and FS in the THSWD group were improved, with significant differences noted between the THSWD group and MI group (Figures 1(c), and 1(d)). In comparison with the animals in the MI group, rats receiving THSWD displayed a tendency of a lower LVEDV value; however, no significant differences were observed between the THSWD group and the MI group. However, the LVESV value in the THSWD group was significantly reduced compared with the value in the MI group (Figures 1(e), and 1(f)). As the EF value is calculated by (LVEDV-LVESV)/LVEDV, the lower the LVESV, the higher the EF reflected. Studies have also reported that the left ventricle can discharge more blood after THSWD treatment. Although the FS value was determined by left ventricular end-systolic (LVESD) and end-diastolic diameter (LVEDD), LVESV was related to LVESD. Therefore, the significant decrease in LVESV contributes to the systolic function of the left ventricle.

### 3.2. Effect of Taohong Siwu Decoction on Infarct Size and Collagen Deposition in the Infarcted Area

**Figure 2:**
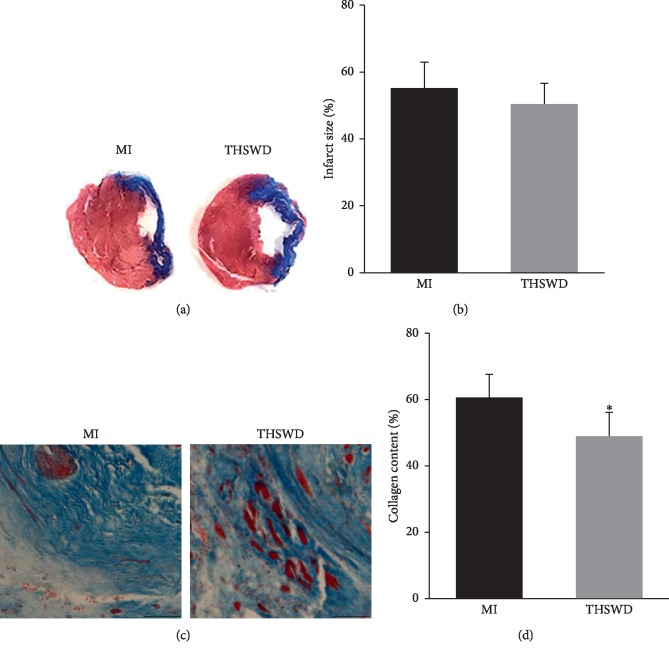
Effect of THSWD on infarct size and collagen deposition in the infarcted area. (a, b) Masson's trichrome staining was performed to detect the infarction size and collagen content in each group. Scale bar = 50 *μ*m. (c) Quantitative analysis of myocardial infarct area. (d) Quantitative analysis of collagen content in the infarcted area. ^*∗*^*P* < 0.05 versus the MI group.

The infarct size and collagen content in the infarct region were determined using Masson's trichrome staining. The left ventricular myocardium was extensively replaced by fibrosis, and severe left ventricular wall thinning was observed both in the MI and THSWD groups. THSWD tended to reduce the myocardial infarct size, with no reported statistical differences observed between the THSWD group and the MI group (Figures 2(a), and 2(b)). Only a small number of viable myocardia was scattered in the infarcted area. Notably, the collagen content in the infarct region of the THSWD group was significantly decreased compared with that in the MI group (Figures 2(c), and 2(d)).

### 3.3. Taohong Siwu Decoction Promoted Angiogenesis after MI

**Figure 3:**
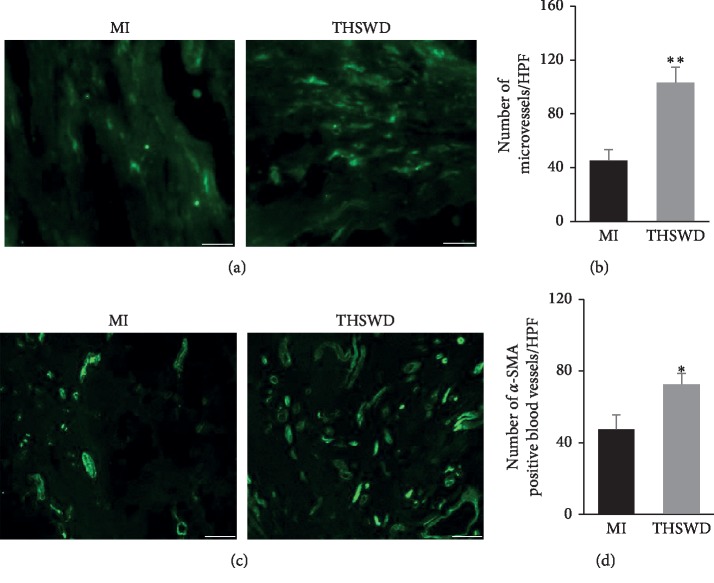
THSWD promoted angiogenesis in the peri-infarction and infarction area of rat hearts after MI. The microvessel in the peri-infarction and infarction area of rat hearts were examined by immunofluorescence staining for CD31 (a). Scale bar = 25 *μ*m and quantified (b). The *α*-SMA-positive blood vessels were detected by immunofluorescence staining for *α*-SMA (c). Scale bar = 50 *μ*m and quantified (d). ^*∗*^*P* < 0.05 and ^*∗∗*^*P* < 0.01 versus the MI group.

The blood perfusion of the lesion after MI is an important factor to evaluate the beneficial effect after treatment. The blood vessel density in the peri-infarction and infarction area of rat hearts was detected using immunofluorescence staining. Compared with the MI group, the administration of THSWD significantly elevated the number of CD31-positive microvessels and increased the number of *α*-SMA-positive blood vessels (Figures 3(a)–3(d)).

### 3.4. Effect of Taohong Siwu Decoction on Cytokines in the Infarcted Area

**Figure 4:**
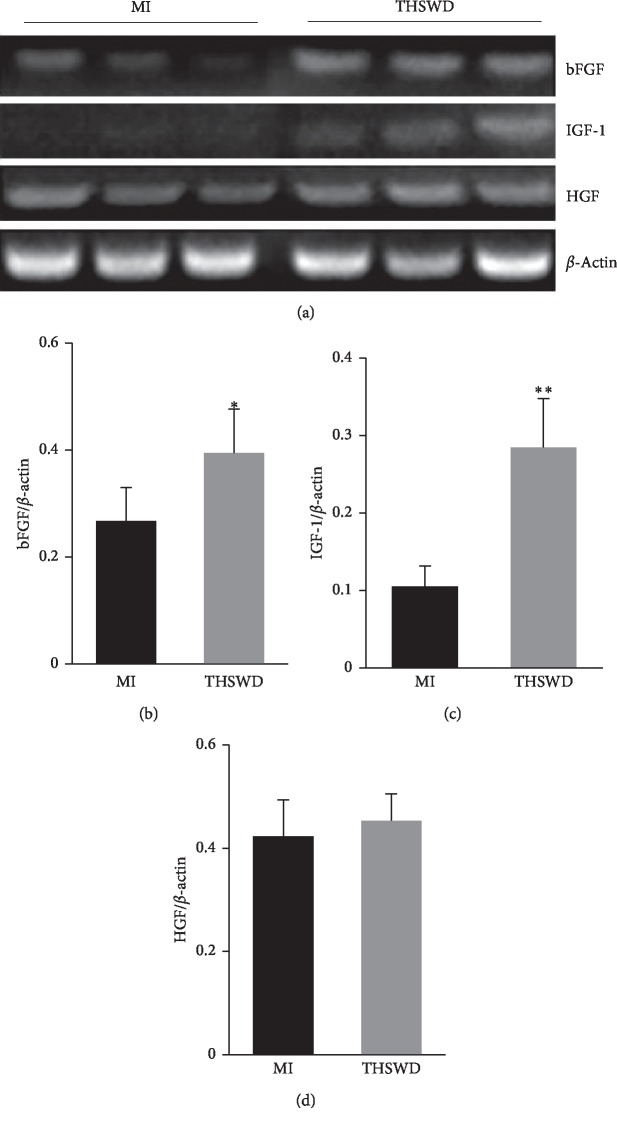
Effect of THSWD on the expression of cytokines in the infarcted area. (a) The mRNA expression of bFGF, IGF-1, and HGF was detected by RT-PCR assay. (b–d) The expression of bFGF, IGF-1, and HGF was calculated and analyzed. ^*∗*^*P* < 0.05 and ^*∗∗*^*P* < 0.01 versus the MI group.

To understand the effect of THSWD on the myocardial microenvironment after MI, the mRNA expression of several cytokines including bFGF, IGF-1, and HGF, which are helpful for survival and neovascularization of the infarcted heart tissue, was evaluated by RT-PCR assay. THSWD significantly increased the expression of bFGF and IGF-1 compared with that in the MI group. No statistical difference was observed in the expression of HGF between the two groups (Figures 4(a)–4(d)).

### 3.5. Attenuation of Apoptosis and Activation of the Akt Signaling Pathway by Taohong Siwu Decoction

**Figure 5:**
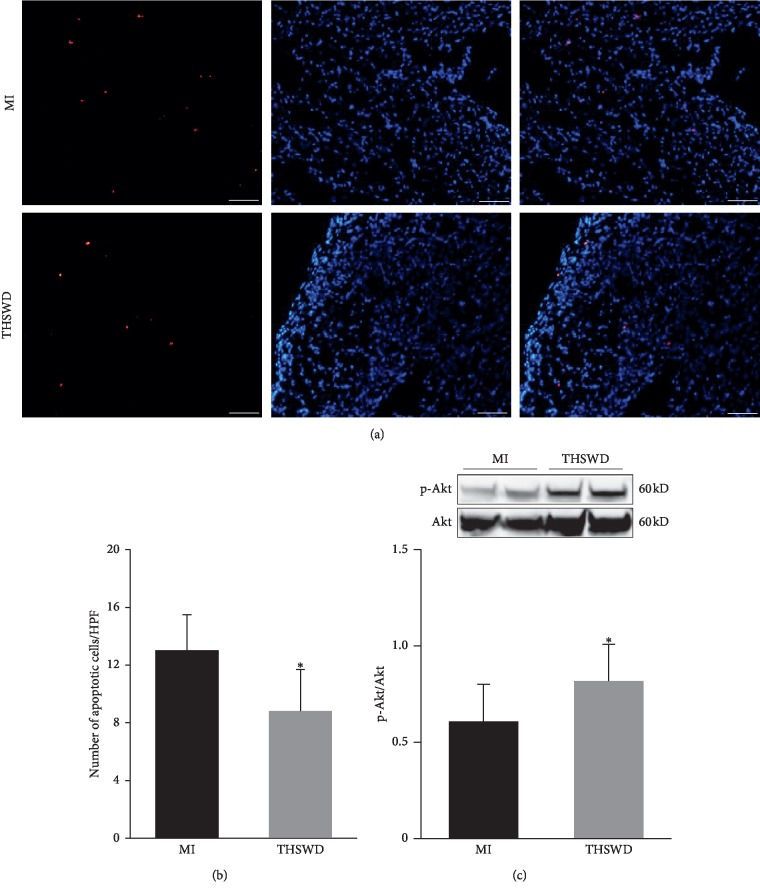
THSWD reduced cell apoptosis and activated the Akt signaling pathway in the myocardial tissue of MI rats. (a) The cell apoptosis 7 days after THSWD treatment was determined by TUNEL staining. (b) The total number of TUNEL-positive nuclei were counted and analyzed. (c) The level of p-Akt in the myocardial tissue was detected using the western blot assay. ^*∗*^*P* < 0.05 versus the MI group.

As cell apoptosis was most prominent during the first week after MI, we assessed cell apoptosis using TUNEL on day 7 after THSWD treatment. TUNEL staining indicated that the administration of THSWD decreased the number of apoptotic cells in the infarcted area (Figures 5(a), and 5(b)). AKT is one of the most actively studied signaling pathways in basic research and drug development as it regulates fundamental cellular functions such as proliferation, growth, transcription, translation, and survival. According to the western blot assay, the level of p-Akt in the myocardial tissue of the THSWD group was significantly higher than that in the MI group, indicating that the Akt signaling pathway could be activated by THSWD in the myocardial tissue post-MI (Figure 5(c)). Overall, the reduced apoptosis could be related to the enhanced p-Akt, which may be mediated by the overexpression of IGF-1 in the THSWD group.

Additionally, THSWD was administrated to normal rats (no MI). THSWD did not alter cardiac function in normal rats, and there were no significant differences in the number of cardiac microvessels between normal rats and normal rats administered THSWD. Furthermore, although THSWD increased the level of p-Akt in the normal heart, no significant difference was observed between rats treated or untreated with THSWD ([Supplementary-material supplementary-material-1]).

### 3.6. Effect of Taohong Siwu Decoction on Mitochondrial Function, Dynamics, and Mitophagy

**Figure 6:**
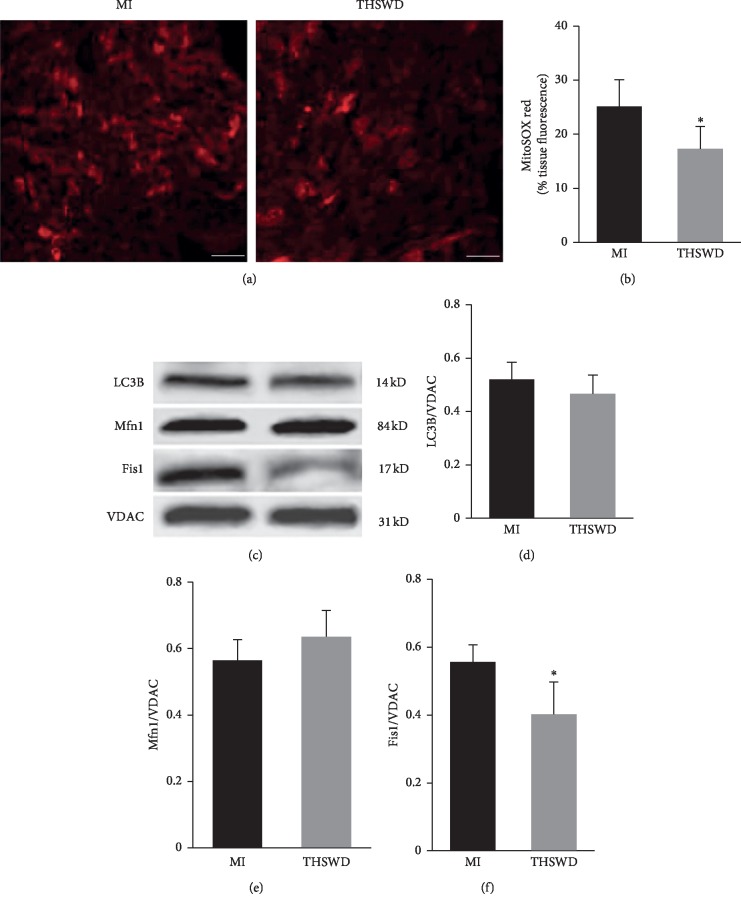
Effect of THSWD on mitochondrial function, dynamics, and mitophagy. (a) The mitochondrial ROS production was measured by MitoSOX staining 7 days after THSWD treatment. Scale bar = 25 *μ*m. (b) The level of MitoSOX red in the myocardial tissue was analyzed. (c) The protein expression levels of mitochondrial LC3B, Mfn1, and Fis1 were detected by western blot. (d–f) The semiquantitative data of western blots for LC3B, Mfn1, and Fis1. ^*∗*^*P* < 0.05 versus the MI group.

Next, we evaluated whether THSWD could exert a beneficial effect on cardiac function by possibly regulating mitochondrial function, dynamics, and mitophagy during the early stage of MI. Mitochondrial damage and ROS production, which are related to cell apoptosis, were also detected on day 7 after THSWD treatment. The mitochondrial ROS production, measured by MitoSOX staining, was significantly reduced in the THSWD-treated hearts compared with that in the saline-treated hearts (Figures 6(a), and 6(b)). LC3B was slightly decreased in the THSWD group compared with that in the MI group. The potential effects of THSWD on mitochondrial dynamics were investigated in terms of mitochondrial fusion and mitochondrial fission, regulated by protein Mfn1 and Fis1, respectively. Our results demonstrated no significance on the expression of Mfn1 between the THSWD group and the control group. However, there was a statistically significant decrease in Fis1 in the THSWD group compared with that in the control group (Figures 6(c)–6(f)).

## 4. Discussion

With a history of more than 3000 years, TCM has a plethora of herbal prescriptions to treat cardiovascular diseases including MI [8]. Despite the lack of evidence demonstrating its advantages and disadvantages, the early use of intravenous injections of Traditional Chinese Medicine in the treatment of acute MI is growing in China [18]. TCM therapy reportedly decreased the blood stasis syndrome scores and the rehospitalization rate during the 6-month follow-up in angina [19]. It has been revealed THSWD combined with conventional treatments demonstrated a potential benefit in relieving angina pectoris without adverse events [16]. However, there is an urgent need to define the effects of TCM therapy in MI. In the formula of THSWD, *Semen Persicae* and *Flos Carthami* are the reported main components involved in blood circulation in order to dissipate blood stasis and dredge the meridians. They significantly analyzed the prolonged thrombin time and thromboplastin time, increased prothrombin time, and lowered fibrinogen content [20]. *Angelica Sinensis* and *Rhizoma Chuanxiong* increased myocardial blood flow, increased oxygen supply, and maintained the myocardial oxygen balance. Furthermore, the effect of VEGF expression in MI rats promoted endothelial cell proliferation and stimulated the number of vessels [21]. Angelica sinensis polysaccharide, a major bioactive component of *Angelica sinensis*, attenuated hypoxia-induced H9c2 cell injury possibly through the downregulation of miR-22 expression [22]. The spectrum-activity relationship indicated that the effective components of *Ligusticum Chuanxiong Hort* had a protective effect on myocardial ischemia [23]. Paeoniflorin, shown to be a bioactive component of *Radix Paeoniae Alba*, significantly reduced the levels of the inflammatory cytokines, including IL-1*β* and TNF-*α* [24]. It was reported that *Radix Rehmanniae Praeparata* was a new potential herbal medicine in the treatment of cardiovascular diseases possibly via the regulation of the PI3K-Akt signaling pathway [25]. In this study, we observed that THSWD increased the EF and FS values after four weeks of treatment, with no significant differences observed between the THSWD group and model group. However, according to the difference between the values after 4 weeks of treatment and those at the baseline, EF and FS in the THSWD group increased significantly. The TCM syndrome typing of MI is *Qi* stagnation and blood stasis, as well as *Yang* deficiency and cold coagulation and *Qi* stagnation and phlegm obstruction. *Qi* stagnation refers to stagnation of the circulation of vital energy and *Yang* deficiency refers to a lack of vital energy. The main role of THSWD includes *Qi* replenishing and activating blood circulation. Hence, on the basis of this formula, further studies are necessary to investigate whether for the recovery of cardiac function in MI rats it will be more conducive to combine THSWD with other TCM with the properties of warming *Yang* and removing phlegm and whether THSWD demonstrated a dose-dependent effect on cardiac function in the treatment of MI. Additionally, the safety of natural products is always a major concern internationally. No obvious adverse events were reported in most THSWD clinical trials. The combination of THSWD with conventional therapy to treat patients with angina pectoris resulted in headaches in the combination group and control group [16]. Moreover, other adverse events associated with THSWD may include nausea, diarrhea or abdominal pain. Therefore, the side effects of THSWD should be monitored during the clinical application.

Angiogenesis is a complicated process consisting of endothelial cell proliferation, directional migration, extracellular matrix remodeling, and vessel maturation [26]. Angiogenesis plays an important role in preserving cardiac functions after MI, recovering oxygen supply, and rescuing the cardiomyocytes from apoptosis and necrosis. The results of this study demonstrated that the administration of THSWD increased the number of CD31-positive microvessels and *α*-SMA-positive blood vessels, suggesting that THSWD may enhance angiogenesis after MI. A previous study reported that the *Flos Carthami* whole extract increased angiogenesis in human microvascular endothelial cells *in vitro* and in zebrafish *in vivo* through multiple mechanisms [27]. *Angelica Sinensis* has reportedly played an important role in angiogenesis and antiapoptosis in the rat cerebral ischemia-reperfusion injury by activating the p38MAPK/HIF-1*α*/VEGF-A signaling pathway [28] and promoting angiogenesis through p38 and JNK 1/2 phosphorylation [29]. In contrast, *Angelica Sinensis* attenuated angiogenesis under certain conditions such as cancer [30]. Therefore, *Angelica Sinensis* may possess a dual regulatory role in the regulation of angiogenesis depending on the different microenvironments. Although THSWD increased the number of blood vessels in the peri-infarction and infarction areas, the perfusion of the ischemic muscle needs to be further investigated by techniques such as laser Doppler analysis.

Over the past decade, cytokine-based therapies have emerged as promising noninvasive treatments in postinfarct cardiac failure and chronic ischemia, stimulating the proliferation and differentiation of endothelial cells and endogenous stem cells and mobilizing these cells toward the ischemic tissue. IGF-1 plays an important role in cellular survival and growth by binding to its specific receptor. A one-time, low-dose IGF-1, in the postreperfusion phase of a large MI, has been shown to translate into long-term preservation of the myocardial cell structure and function [31]. Local myocardial IGF-1 delivery with biotinylated peptide nanofibers reduced the post-MI infarct size and LV dysfunction in rats [32]. In addition, post-MI, treatment with IGF-1 significantly induced angiogenesis [33]. bFGF is one of the most potent growth factors known to promote proliferation, migration, and survival of cells and induce neovascularization [34]. Therapeutic angiogenesis induced by bFGF using biodegradable gelatin hydrogel sheets was safe, increased vascular density, and improved LV systolic function in canine chronic MI models [35]. In the lung, bFGF has been known to decrease pulmonary fibrosis and inhibit fibroblast collagen production and myofibroblast differentiation [36]. In the present study, we observed that following the intragastric administration of THSWD, the expression of IGF-1 and bFGF in the myocardial tissue of MI rats was higher in the THSWD group than that in the model group. Therefore, THSWD may decrease the collagen content and promote angiogenesis partly by upregulating the expression of IGF-1 and bFGF. Moreover, both IGF-1 and bFGF could activate the PI3K/Akt pathway to attenuate myocardial apoptosis after myocardial injury [32, 37]. We observed that THSWD could significantly increase the level of p-Akt, which may be related to the elevated expression of IGF-1 and bFGF. However, the protein levels of IGF-1 and bFGF need to be measured. Furthermore, the underlying molecular mechanisms of THSWD enhanced IGF-1 and HGF secretion needs to be elucidated.

Mitochondrial ROS production represents one of the major determinants of infarct size and heart remodeling during cardiac ischemia. The damaged mitochondria can be eliminated by macroautophagy or mitophagy. In our study, THSWD slightly decreased the expression of LC3B, a key component of the phagophore that engulfs the damaged mitochondria. Because the timing and magnitude of activation are variable, activation of autophagy mechanisms in cardiomyocytes could be beneficial or maladaptive [38]. It is also well known that excessive mitochondrial ROS production results in the impairment of the mitochondrial dynamic processes. THSWD significantly decreased the expression of Fis1 which regulates mitochondrial fission. It has been reported that 12 and 18 weeks after coronary artery ligation, Fis1 was increased by 80% and 31%, respectively [39]. Acute inhibition of excessive mitochondrial fission may produce long-term benefits after acute MI [40]. However, the expression levels of other mitochondrial fission/fusion proteins, in the MI heart after THSWD treatment, need to be further clarified.

In this study, THSWD only slightly improved cardiac function 4 weeks after treatment. Stem cells possess the ability to proliferate for an extended period and differentiate into specific cell types under appropriate conditions. Thus, they hold great innovative potential in regenerative medicine and tissue engineering [41]. We have observed that transplantation of mesenchymal stem cells (MSCs) reduced scar size and cell apoptosis as well as improved cardiac function after MI [42, 43]. Nevertheless, the efficacy of stem cell therapy is hampered by a low survival and differentiation rate in the hostile microenvironment due to myocardial ischemia and oxygen deficit [44]. We proposed that THSWD could ameliorate the microenvironment after MI as it not only raised the expression of IGF-1 and bFGF but also enhanced angiogenesis and activated the PI3K/Akt signaling pathway. Recent evidence suggested that Danhong injection enhanced the residence of MSCs in the cardiac tissue, promoted angiogenesis, and reduced myocardial infarct size [45]. We also found that Guanxin Danshen formulation enhanced the effects of MSC transplantation for the treatment of MI [46]. Thus, it remains worthwhile to investigate the effects of THSWD combined with the transplantation of MSCs for the treatment of MI in the future.

## 5. Conclusion

In summary, our current study demonstrated that THSWD could slightly improve the cardiac function after MI, but significantly increased the expression of IGF-1 and bFGF, reduced collagen deposition, promoted angiogenesis, reduced the cell apoptosis, activated PI3K/Akt signaling pathway, and decreased the mitochondrial ROS production and Fis1 expression. Collectively, THSWD may have a beneficial effect on cardiac function probably by improving the local hostile microenvironment and decreasing mitochondrial fission, offering a promising adjunct treatment for MI.

## Data Availability

The data used to support the findings of this study are available from the corresponding author upon request.

## Abbreviations

TCM: Traditional Chinese Medicine
THSWD: Taohong Siwu decoction
MI: Myocardial infarction
EF: Ejection fraction
FS: Fractional shortening
LVESV: Left ventricular end-systolic volume
LVEDV: Left ventricular end-diastolic volume
PBS: Phosphate buffer solution
bFGF: Basic fibroblast growth factor
IGF-1: Insulin-like growth factor-1
HGF: Hepatocyte growth factor.

## Supplementary Materials

Supplementary Materials FIG. S1: the effects of THSWD on a normal heart. (A) THSWD did not affect the cardiac function of normal rats. (B) There was no significant difference in the number of microvessels in the hearts between normal rats received THSWD and rats which did not receive THSWD. (C) THSWD tended to increase the level of p-Akt in the normal heart, but the difference was not significant.
