# Supplementary material for: Taohong Siwu Decoction Exerts a Beneficial Effect on Cardiac Function by Possibly Improving the Microenvironment and Decreasing Mitochondrial Fission after Myocardial Infarction
Source: Cardiol Res Pract. 2019 Dec 10;2019:5198278. doi: 10.1155/2019/5198278 (PMC6925791; doi:10.1155/2019/5198278)
Supplement: Supplementary Materials — FIG. S1: the effects of THSWD on a normal heart. (A) THSWD did not affect the cardiac function of normal rats. (B) There was no significant difference in the number of microvessels in the hearts between normal rats received THSWD and rats which did not receive THSWD. (C) THSWD tended to increase the level of p-Akt in the normal heart, but the difference was not significant. [file 5198278.f1.docx]

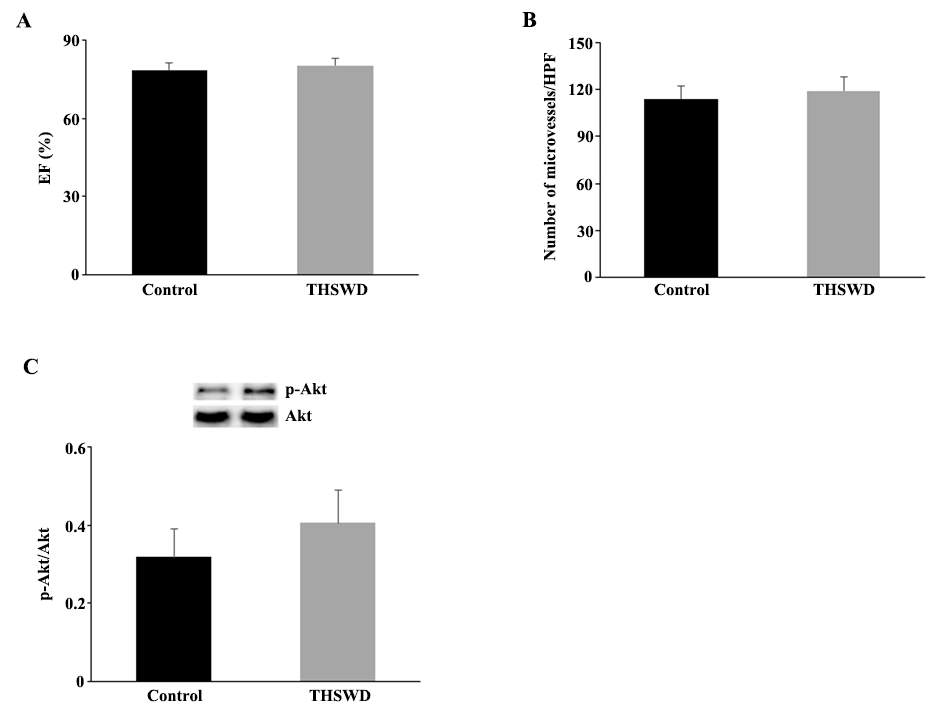


FIG. S1. The effects of THSWD on normal heart. (A) THSWD did not affect the cardiac function of normal rats. (B) There was no significant difference in the number of microvessels in the hearts between normal rats received THSWD or not. (C) THSWD tended to increase the level of p-Akt in the normal heart, but the difference was not significant.
